# Art is In: Preliminary Results of a Remotely Delivered Therapeutic Arts Intervention on Patients with Dementia and their Caregivers

**DOI:** 10.1002/alz70858_107123

**Published:** 2025-12-26

**Authors:** Prachi Shah, Fabiana Goodman, Kristen Taruc, Tessa Garcia McEwen, James Mastrianni, Elena Badillo‐Goicoechea, Kaitlin Seibert

**Affiliations:** ^1^ The University of Chicago Pritzker School of Medicine, Chicago, IL, USA; ^2^ Goldmind Arts, Chicago, IL, USA; ^3^ The University of Chicago, Chicago, IL, USA; ^4^ University of Chicago, Chicago, IL, USA

## Abstract

**Background:**

Therapeutic art is a common activity for patients with dementia (PWD) and their caregivers, but accessibility to dementia‐friendly programs is a barrier. In response to the COVID pandemic, we adapted an in‐person “Art Is…” therapeutic art class into an 8‐week dyadic, fully remote program and measured the impact on patient behavior, caregiver burden, and patient‐caregiver relationship.

**Method:**

In a double crossover design, participant dyads (one PWD and their caregiver) were assigned to two groups, intervention and control. For the intervention period, dyads were mailed weekly kits over an 8‐week period. All patients completed an 8‐week control period of standard care, either before or after the intervention period. Assessments measuring patient symptom severity, patient‐caregiver relationship quality, and caregiver burden were administered at enrollment, at 8 weeks and at 16 weeks. Mixed effect regression models with patient random effects and interaction effects by subgroup (dementia subtype and caregiver) were used to analyze differences between each outcome in the intervention period as compared to the control period.

**Result:**

A total of 66 dyads were enrolled into the study, 44 of which completed the study. Attrition was largely attributable to caregiver responsiveness and availability for assessments. However, remote delivery of the therapeutic art program was feasible in our study population. Although there were no overall significant differences between the pre‐intervention and post‐intervention assessments, interaction analyses indicated there was a significant decrease in verbal aggressive behaviors for patients with mild cognitive impairment or atypical dementia (*p* = 0.01, *p* = 0.02). Additionally, when the patient's child was the primary caregiver, there was a significant improvement in a caregiver's feelings of accomplishment (*p* = 0.05) and a decrease in verbal aggressive behaviors (*p* = 0.04). A limitation of our study is the small sample size.

**Conclusion:**

This program demonstrates that at‐home therapeutic arts programs are a feasible alternative to in‐person programs. Future, higher powered analyses are required to assess efficacy of therapeutic art interventions for mood and behavior in PWD, caregiver burden and patient‐caregiver relationships.

